# The impact of human and livestock respiration on CO_2_ emissions from 14 global cities

**DOI:** 10.1186/s13021-022-00217-7

**Published:** 2022-11-03

**Authors:** Qixiang Cai, Ning Zeng, Fang Zhao, Pengfei Han, Di Liu, Xiaohui Lin, Jingwen Chen

**Affiliations:** 1 State Key Laboratory of Numerical Modeling for Atmospheric Sciences and Geophysical Fluid Dynamics, Institute of Atmospheric Physics, Chinese Academy of Sciences, Beijing, China; 2 Department of Atmospheric and Oceanic Science, and Earth System Science Interdisciplinary Center, University of Maryland, College Park, MD USA; 3 Key Laboratory of Geographic Information Science (Ministry of Education), School of Geographic Sciences, East China Normal University, Shanghai, China; 4 State Key Laboratory of Atmospheric Boundary Layer Physics and Atmospheric Chemistry, Institute of Atmospheric Physics, Chinese Academ y of Sciences, Beijing, China; 5 State Key Laboratory of Hydrology- Water Resources and Hydraulic Engineering, Hohai University, Nanjing, China

**Keywords:** Human respiration, Livestock respiration, City CO_2_ emission, Urban metabolism

## Abstract

**Background:**

The CO_2_ released by humans and livestock through digestion and decomposition is an important part of the urban carbon cycle, but is rarely considered in studies of city carbon budgets since its annual magnitude is usually much lower than that of fossil fuel emissions within the boundaries of cities. However, human and livestock respiration may be substantial compared to fossil fuel emissions in areas with high population density such as Manhattan or Beijing. High-resolution datasets of CO_2_ released from respiration also have rarely been reported on a global scale or in cities globally. Here, we estimate the CO_2_ released by human and livestock respiration at global and city scales and then compare it with the carbon emissions inventory from fossil fuels in 14 cities worldwide.

**Results:**

The results show that the total magnitude of human and livestock respiration emissions is 38.2% of the fossil fuel emissions in Sao Paulo, highest amongst the 14 cities considered here. The proportion is larger than 10% in cities of Delhi, Cape Town and Tokyo. In other cities, it is relatively small with a proportion around 5%. In addition, almost 90% of respiratory carbon comes from urban areas in most of the cities, while up to one-third comes from suburban areas in Beijing on account of the siginificant livestock production.

**Conclution:**

The results suggest that the respiration of human and livestock represents a significant CO_2_ source in some cities and is nonnegligible for city carbon budget analysis and carbon monitoring.

**Supplementary Information:**

The online version contains supplementary material available at 10.1186/s13021-022-00217-7.

## Background

**Fig. 1:**
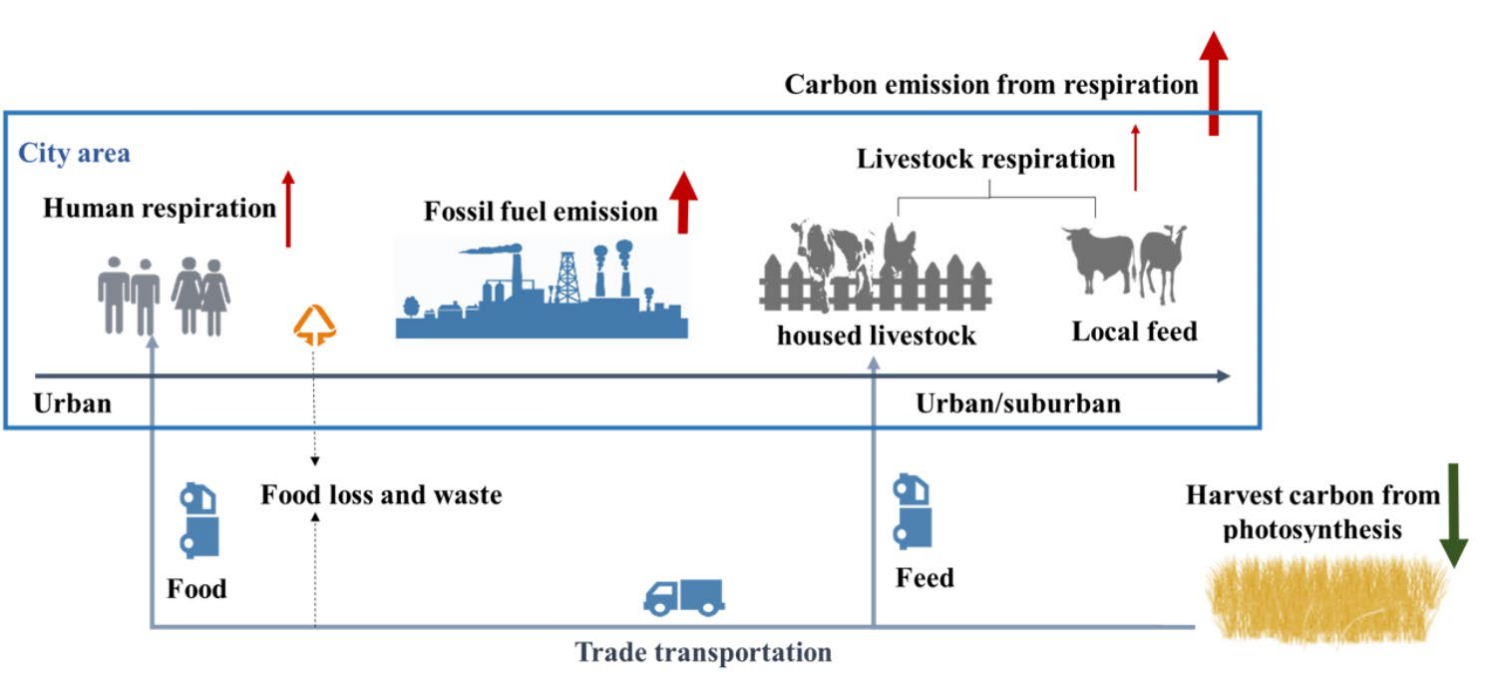
The process of urban material metabolism with crop production, the digestion of food and feedstuff by humans and livestock, and finally the release of carbon to the atmosphere via respiration

Currently, approximately 55% of the world’s population [1] occupies only 0.37% of the global land surface [2, 3]. From the viewpoint of the process of urban material metabolism, the total harvested carbon transported into an urban system releases CO_2_ through the processes of human and livestock metabolism and constitutes a part of the global CO_2_ cycle (Fig. 1) [4–7].

However, CO_2_ from human and livestock respiration is often neglected due to its perceived small magnitude compared to fossil fuel emissions (FFE) from the burning of fuels for electricity, heating and industrial purposes, other industrial processes and ground transportation within city boundaries [8, 9]. Some researchers have considered human respiration as a significant contribution only in street level or residential areas, where CO_2_ emissions from power plants and industry can be ruled out [10–12]. Others have regarded biogenic emissions to be a nonnegligible contribution at night or even during winter [13–16] but have not included CO_2_ release from humans and livestock respiration (HLR).

However, human and livestock respiration could be important in cities with high demands for food and feedstuff consumption. It has been estimated that, human and livestock respiration could represent 1.2 − 30% of FFE in densely populated regions (Table 1), such as some typical cities of Beijing, Greater Paris and Mexico City [17–23]. When comparing CO_2_ emissions on regional-scales from both bottom-up inventories and top-down approaches, human and livestock repiration is a significant component in the reconciliation of the differences [23]. Thus, accurately estimating the amount of HLR could improve the results of atmospheric CO_2_ flux inversion approach for estimating FFE and for comparison with the bottom-up technique [21, 23, 24]. However, the published studies (in Table 1) only focus on individual cities, and the HLR has been clearly calculated in only a few cities. Moreover, only the studies of Zhao et al. and Gurney et al. include livestock respiration [22, 23], while the CO_2_ release from respiration in other studies is only from humans. Thus, in areas with high population density, detailed estimation of the HLR is necessary for CO_2_ monitoring and CO_2_ flux inversion. However, a high-resolution dataset of HLR has rarely been reported on a global scale or in cities globally.

**Table 1:** Published results for CO_2_ releases from human and livestock respiration compared with total fossil fuel emissions

City	Respiration to fossil fuel proportion (%)	Reference
Phoenix, AZ, USA	1.9%^1^	Koerner and Klopatek, 2002
Beijing, China	30%	Ciais et al., 2007
Shanghai, China	12%	
Chicago, Illinois, USA	1.2%	West et al., 2009
Mexico City, Mexico	6.4%^1^	Velasco and Roth, 2010
Greater Paris, France	8%	Bréon et al., 2014
Marion County, Indiana, USA	2.9%	Gurney et al., 2017
Nanjing, China	6.8%^1^	Zhao et al., 2014

^1^ The proportion is not given directly in the reference, but converted from their results. The proportion in Mexico is converted from the respiration contribution to total CO_2_ emissions to the respiration to fossil fuel. The proportion in Phoenix and Nanjing are calculated based on CO_2_ emissions from different sources

The purpose of this study is to establish high-resolution datasets of global human and livestock carbon production and to compare with CO_2_ from FFE within large cities/metropolitan areas around the world. Excluding food loss/waste and livestock feed from local sources, the harvest carbon from crop production should correspond to the total human and livestock consumption of carbon. The fourth section discusses these carbon budgets and presents an uncertainty analysis.

## Methods

### Study area and in-boundary FFE

The 14 reported global major cities according to the research of Chen et al. [25] are selected as the study area and include Bangkok, Beijing, Shanghai, Delhi, Cape Town, Sao Paulo, Tokyo, Greater Paris, Greater London, Los Angeles, Manhattan, New York City, Washington D.C., and Greater Toronto (see Table 2 and Additional file 1: Figure S1). The definitions of the 14 cities ranges from ‘district’ to ‘metropolitan’ (see also Table 2) [26]. The CO_2_ from human and livestock respiration is directly emitted within city boundaries, which is belongs to the scope 1 for direct emissions, that is, produced in city boundaries mainly from fossil fuel combustion, transportation, industrial processes and production, land use and waste [27]. To compare the CO_2_ emissions from human and livestock respiration within these global cities, we retrieved the in-boundary anthropogenic FFE from Chen et al. [25], who estimated the total FFE directly within the city boundaries of these 14 cities and metropolitan areas around the world.

**Table 2:** The *HR*_*h*_ of 6 age-sex groups in 7 global regions (unit: kg C yr^− 1^)

Region	ages 0–9, female	ages 0–9, Male	ages 10–19, female	ages 10–19, Male	ages 20+, female	ages 20+, Male
South Asia	55.2	55.2	77.6	83.0	99.5	94.8
East Asia & Pacific	63.7	63.7	85.7	92.9	105.3	101.6
Sub-Saharan Africa	60.5	58.1	83.4	86.8	105.3	94.8
Latin America & Caribbean	65.3	66.5	86.9	94.8	106.9	100.6
Europe & Central Asia	66.5	66.5	88.1	97.6	109.5	101.6
Middle East & North Africa	63.7	63.7	87.6	92.9	101.5	94.1
North America	68.9	68.9	90.2	102.0	105.3	105.5

Our study also separated city areas into two subcategories, urban and suburban. The urban extent of each city is based on the 1:10 m urban areas shapefile from Nature Earth (https://www.naturalearthdata.com), which is derived from 2002 to 2003 MODIS satellite data at 1 km resolution [28]. Urban areas are defined as built-up areas with high population densities, high radiance levels in commercial/industrial areas and high-density residential land cover, instead of being based on impervious surfaces [29]. The suburban refers to the remaining area within the administrative boundary of the city that is not included in the built-up area.

### Estimate methods for HLR

The CO_2_ released from respiration of per person (*HR*_*h*_) or per head of livestock (*LR*_*l*_) is obtained according to the basal metabolic rate (BMR). The BMR refers to the minimum level of energy required to sustain vital functions of organs at complete rest in a neutrally temperate environment and in a fasting state. It is measured by heat production or oxygen consumption and can be expressed as Cal m^− 2^ h^− 1^, Cal kg^− 1^ h^− 1^ or O_2_ g^− 1^ h^− 1^ for individuals [30, 31]. For various mammals, the oxygen consumption rate per body mass consistently decreases with increasing body size, while the rate of oxygen consumption for individuals against body mass tends to decrease along regression lines in logarithmic coordinates (poultry have a similar equation to mammals) [32]. Additionally, oxygen is combined with carbon according to the respiration reaction. Therefore, based on the BMR of each species, we can estimate the CO_2_ produced by respiration according to the oxygen consumption. What’s more, concerning the metabolic enhancement caused by exercise metabolism and other factors, the physical activity level (PAL) was defined in terms of three levels of physical activity [33]. For simplicity, we assume that the WHO recommended PAL = 1.55 could be used as an uniform parameter for global countries and for different gender and age groups for both human and livestock [34].

### The method of *HR*_*h*_

In this study, the BMR is given by 6 age-sex groups and 7 global regions (Additional file 1: Table S1), which was obtained according to the body mass of each age-sex group in each region and the daily BMR predicted by the FAO for different age groups and for both sexes [35]. Then we convert the BMR in the unit of heat production (MJ day^− 1^) into oxygen consumption (L O_2_ day^− 1^, see Additional file 1: Table S2) by introducing the thermal equivalent of oxygen (20.2 kJ L^− 1^). Finally, the *HR* in age-sex group *h* (*HR*_*h*_) is convert as carbon release in kg C yr^− 1^ (Table 3). The equation of *HR*_*h*_ could be:

1 $$H{R_h} = \frac{\begin{array}{l}BM{R_h} \times Body\_weigh{t_h} \times \frac{{M\left( {{O_2}} \right)}}{{{V_m}}}\\\times \frac{{12}}{{32}} \times 24 \times 365\end{array}}{{{{10}^6}}}$$

2 $${Body\_weight}_{h}={BMI}_{h}\times {{Body\_height}_{h}}^{2}$$

**Table 3:** The parameters of eight types of livestock

Livestock	BMR (kg O_2_ yr^− 1^)	*LR*_*l*_^1^ (kg C yr^− 1^ per head)	Global total production (million head in 2010)^2^	References
horses	813.43	305.04	59.66	M. A. Elgar and P. H. Harvey (1987)
pigs	103.24	19.36	974.41	
cattle and buffalo	578.66	217.00	1603.86	
goats	85.60	32.10	910.83	
sheep	127.65	47.87	1076.36	
chickens and ducks	26.95	1.25	22311.21	B. M. Freeman (1963)

1. *LR*_*l*_ is the CO_2_ release from respiration of per head of livestock

2. Data comes from FAOSTAT, http://www.fao.org/faostat/en/#home

where *BMR*_*h*_ is the BMR with units of ml O_2_ g^− 1^ h^− 1^; $M\left({O}_{2}\right)$ is the molecular mass of O_2_ in g mol^− 1^; and *V*_*m*_ is the molar volume of gas in 22.4 L mol^− 1^; The *Body_weight*_*h*_ is the body mass estimated as the product of the body mass index (BMI) and the mean height for each age-sex group in each region as formulae (2). The mean body height and mean BMI of each age-sex group in each region are obtained from NCD Risk Factor Collaboration (NCD-RisC) [36, 37] (detail method of the BMR estimation is introduced in Additional file 1:Method). The ratio of carbon (C) and O_2_ is set to 12/32 according to the processes of respiration, which can be expressed by the following chemical equation [38]:

$${(CH}_{2}O)+{O}_{2}\to {H}_{2}O+{CO}_{2}$$

where (CH_2_0) represents the composition of biological material.

### The method of *LR*_*l*_

The BMR (ml O_2_ g^− 1^ h^− 1^) of mammalian livestock and chickens (Additional file 1: Table S4) are measured values from previous experimental results that controlled the environmental temperature, nutrition, age and activity level [39–41]. The amount of *LR* of species *l* (*LR*_*l*_) represents the total carbon released during the days when animals are alive (Table 4). Therefore, we also assumed that the life span of poultry is 42 days, that the life span of pigs is half a year [42, 43], and that all species except poultry and pigs live for more than one year. The *LR*_*l*_ is estimated from the following equation:

3 $$L{R_l} = \frac{{\left( \begin{array}{l}BM{R_l} \times {\rm{Bod}}y\_weigh{t_l} \times \frac{{M\left( {{O_2}} \right)}}{{{V_m}}}\\\times \frac{{12}}{{32}} \times 24 \times 365\end{array} \right)}}{{{{10}^6}}}$$

**Table 4:** Sources of data on humans and livestock

Data	Data source	Resolution	Time range
Gridded Population of the World, Version 3 (GPWv3) [45, 46]	Socioeconomic Data and Applications Center (SEDAC)	2.5 arc-minute regridded to 30 arc-second	1990, 1995
Gridded Population of the World, Version 4 (GPWv4) [45, 46]		30 arc-second	2000, 2005, 2010, 2015
Gridded Livestock of the World (GLW) [47–54]	FAO	5 arc-minutes regridded to 30 arc-second	2010
Livestock production [55]	FAOSTAT	national total	1960–2014

where *BMR*_*l*_ is the BMR with units of ml O_2_ g^− 1^ h^− 1^; *Body_weight*_*l*_ is the average of different breeding ages and genders of each species *l*; $M\left({O}_{2}\right)$, *V*_*m*_ and the ratio of carbon (C) and O_2_ are the same with folume (1) for *HR*_*h*_.

### The method and data source of HLR

In this study, the population and livestock production we use are reported as high-resolution datasets (see Sect. Datasets of humans and livestock). The total HLR is the sum of HR and LR, which are estimated by multiplying the CO_2_ emission of each individual (*HR*_*h*_ and *LR*_*l*_) by the total population/livestock production in each grid within city boundaries. We assume that in the same region, the population in each grid have a unified fraction of each age-sex group. Thus, the HR in each grid is actually a weighted sum of the *HR*_*h*_ in each age-sex group. The formulaes for HR and LR in grid *i* are given as:

2 $$\text{HR}=Population\left(i\right)\times \sum ({f}_{h}\times {HR}_{h})$$

3 $$\text{LR}=\sum \left({Livestock}_{l}{\times LR}_{l}\right)$$

where *Population(i)* is the total population in grid *i*; *f*_*h*_ is the fraction of age-sex group *h* in the region where grid *i* belongs to; *Livestock*_*l*_ is the production of species *l* in grid *i*. The annual fractions for 6 age-sex groups of the total population in 7 regions come from the World Bank [44].

### Datasets of humans and livestock

The HLR in each city are extracted from high-resolution vector datasets (see Table 5). The Gridded Livestock of the World (GLW) datasets include global distributions of eight major livestock species (also see Additional file 1: Table S4). It should be noted that the total cattle and poultry production in Beijing from the high-resolution datasets is 17 times higher than the statistical data from the National Bureau of Statistics of China (NBS, http://data.stats.gov.cn/english/), while cattle production is consistent with census statistics for Shanghai, Delhi and Sao Paulo (Additional file 1: Table S5-S6). As the detailed cattle census statistics of Beijing for GLW were mined from the NBS, we consider the values from official source of China are more reliable. Thus, we first corrected the livestock production in each grid in Beijing according to the spatial distribution from GLW and the total livestock production from NBS.

**Table 5:** Definition, population and carbon emissions of the 14 cities and metropolitan regions in this study

City or metropolitan region	Year	Definition	Area (km^2^)	Population (thousands)	Total in-boundary FFE (Mt C)	Human Respiration		Livestock Respiration	
						Kt C	%	Kt	%
Bangkok	2005	Bangkok Metropolis	1568.45	5659	7.5	**630.4**	**8.4**	3.1	0.04
Beijing	2006	Beijing Municipality	16387.60	15,810	31.4	1943.6	**6.2**	483.6	1.5
Cape Town	2005	City of Cape Town Metropolitan Municipality	2443.63	3497	1.7	**272.1**	**16.3**	9.3	0.6
Delhi	2000	Metropolis	1500.84	13,200	3.5	**1110.6**	**32.1**	118.5	3.4
Greater London	2003	Greater London	1601.83	7364	8.8	730.6	**8.3**	6.1	0.07
Greater Paris	2005	Ile de France	12026.70	11,532	13.7	**1142.5**	**8.3**	43.9	0.3
Greater Toronto	2005	Greater Toronto	7610.32	5556	12.1	**558.9**	**4.6**	35.1	0.3
Los Angeles	2000	County	10587.20	9519	21.2	**944.3**	**4.5**	11.4	0.05
Manhattan	2005	Borough	67.61	1570	2.4	**149.6**	**6.3**	0.1	< 0.01
New York City	2005	City	739.51	8170	11.8	**643**	**5.5**	0.3	< 0.01
Sao Paulo	2011	Municipality	1520.90	11,300	2.9	1105.4	**38.2**	1.7	0.06
Shanghai	2006	Shanghai Municipality	6884.65	18,150	49.1	1943.6	**4.0**	142.4	0.3
Tokyo	2006	Tokyo Metropolis	1804.78	12,678	10.9	1212.8	**11.1**	3	0.03
Washington DC	2000	District of Columbia	177.71	572	1.9	**56.8**	**3.1**	0.5	0.03

1 The population data for Manhattan were adopted from NYC Open Data (https://data.cityofnewyork.us/City-Government/New-York-City-Population-by-Borough-1950-2040/xywu-7bv9), and the population data for the other 13 cities were obtained from Kennedy et al. [56, 57]

2. The human respiration in bold is directly extracted from GPW high-resolution population data, and that in black font is from linear interpolation

3. The values (in %) in bracket show the proportion of human and livestock respiration compared with total FFE.

4. The livestock respiration in Beijing was corrected with livestock production from the National Bureau of Statistics of China

The city boundaries we used in this study come from the database of Global Administrative Areas (GADM) version 2.0 (http://gadm.org/). The shapefile with polygon features of 14 cities was first converted to a high-resolution vector-form dataset at a resolution of 30 arc-second and can be used as a region mask to extract values for population and livestock production within different cities.

To match the years with FFE in 14 cities from Chen et al. [25], we use linear interpolation to obtain the annual human respiration after extracting the total CO_2_ released in each city every 5 years based on GPWv3 and GPWv4. For livestock, the GLW only provides the high-resolution livestock production in 2010. Thus, we assume that the trend of livestock production in each city is the same as that of the country where the city belongs to (data source see Table 5). The total LR in countries was obtained according to the method described in Sect. 2.2. Then, the total annual livestock respiration within cities were estimated from the annual livestock production of country from FAO and the LR of cities in 2010.

## Results

### Global CO_2_ release contributed by human and livestock respiration

We now examine results for the global total CO_2_ contributed from human and livestock respiration. Globally, the annual total HR was increased from 0.52 Gt C in 1995 to 0.68 Gt C in 2015 (approximately 0.64 Gt C in 2010). In terms of global total LR, it was approximately 0.81 Gt C in 2010, with the majority contributed by cattle, followed by sheep and buffalo, accounting for 60.2%, 10.6% and 8.1% of the total livestock carbon, respectively (Additional file 1: Figure S2(b)).

**Fig. 2:**
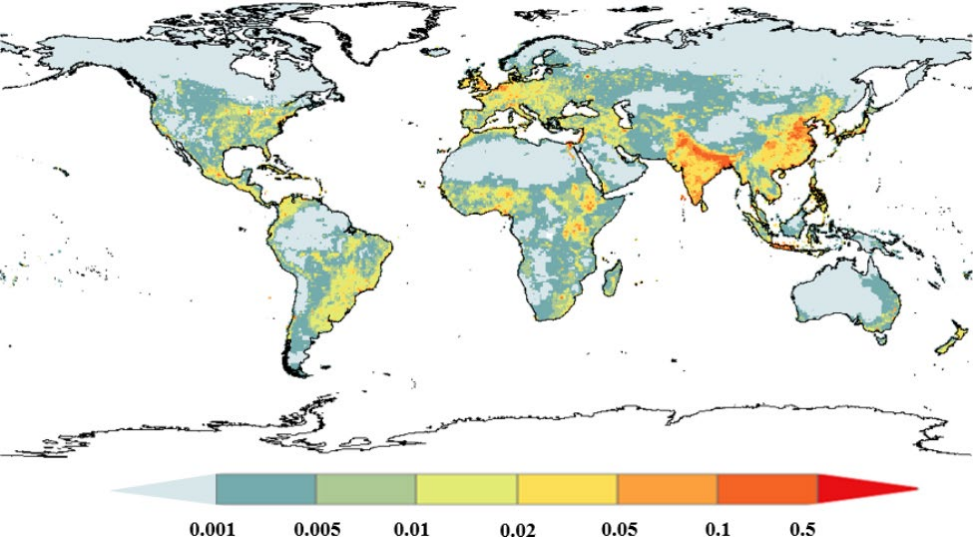
The spatial distribution of total CO_2_ released from human and livestock respiration (HLR) (kg C m^− 2^ yr^− 1^)

The spatial distribution of total HLR is shown in Fig. 2. High values of HLR are found in East Asia, southern Asia and Europe and are mainly contributed by humans (Additional file 1: Figure S2(a)). Meanwhile, the high values found in Australia, New Zealand, South America and central North America are mostly related to livestock respiration (Additional file 1: Figure S2(b)). Moreover, the spatial distribution of human respiration shows a consistent spatial pattern with population because it is simply from population multiplied by the BMR.

### Carbon release from human and livestock respiration in 14 cities

**Fig. 3:**
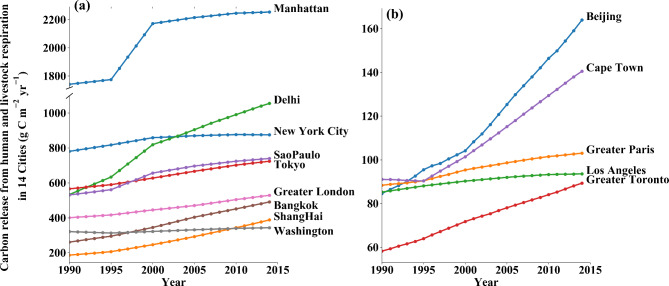
Variation in the total HLR in 14 cities. (a) Greater than 200 g C m^− 2^ yr^− 1^ in from 1990, (b) less than 200 g C m^− 2^ yr^− 1^ befor 2014

Based on the high-resolution gridded data, we extracted the value of HLR in 14 large cities and metropolises. Figure 3 shows the variation of HLR per unit area in the 14 cities. The highest HLR per area in 2014 occurred in Manhattan, followed by Delhi and New York City, with values of 2235.7, 1055.0 and 875.4 g C m^− 2^ yr^− 1^, respectively. Greater Toronto featured the lowest HLR per area, with a value as 89.3 g C m^− 2^ yr^− 1^.

From the viewpoint of the variation trend, all 14 cities have an increasing trend in HLR over the studied 25 years. The CO_2_ emission increased by up to 107.8% with a value of 8.7 g C m^− 2^ yr^− 1^ over 25 year in Shanghai, while Delhi and Beijng increased by 97.8% and 94.0% with the values of 22.6 and 3.4 g C m^− 2^ yr^− 1^, respectively, while the CO_2_ emission only increased 0.7% in Washington DC with a value of 1.3 g C m^− 2^ yr^− 1^. Among the total HLR in 14 cities, human contribute much more than livestock, and the trend of HLR is dominated by the increasing population (see Fig. 3 and S3).

For the values of livestock respiration, the differences among cities are noticeable. The total LR in Beijing (502.0 Kt C yr^− 1^ in 2014) is 4 times those of Delhi (122.0 Kt C yr^− 1^ in 2014) and Shanghai (147.8 Kt C yr^− 1^ in 2014) and might be related to cattle farms in the southern and northeastern portions of Beijing. Among the 14 cities, 9 cities had a descending trend from 1990 to 2014, including Greater Paris, Los Angeles, Cape Town, Tokyo, Bangkok, Greater London, Washington DC, New York City and Manhattan (Additional file 1: Figure S3(b)). Part of the reason for this decline is that, with the development of cities, the livestock industry has gradually shifted to the surrounding areas outside of the city.

**Fig. 4:**
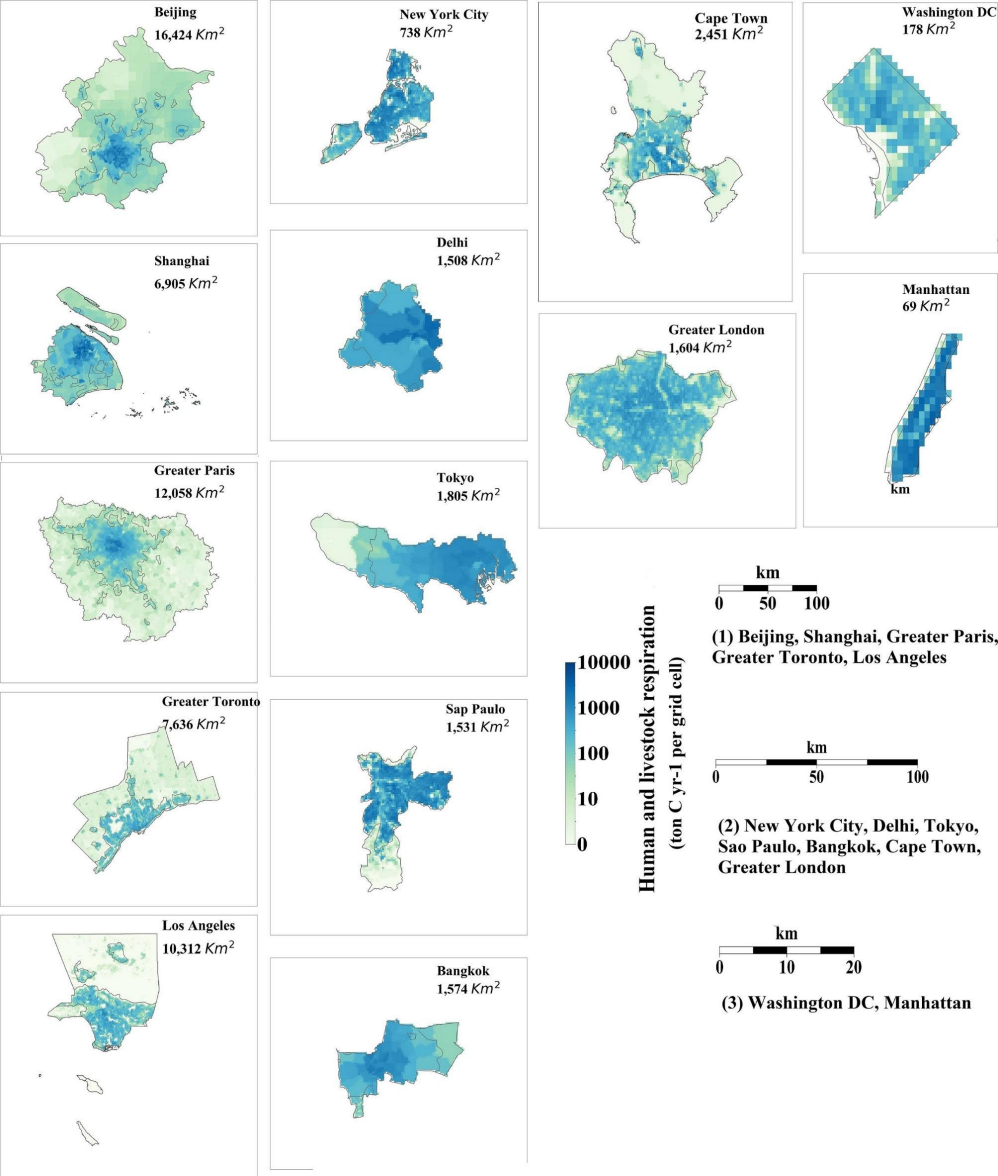
The HLR per in 14 cities and metropolitan regions in 2010 at a resolution of 1 km (ton C yr^− 1^ per grid cell). The areas within the gray line show the urban extent. Three scale bars are included. Scale bar (1) corresponds to Beijing, Shanghai, Greater Paris, Greater Toronto and Los Angeles; scale bar (2) corresponds to New York City, Delhi, Tokyo, Sao Paulo, Bangkok, Cape Town and Greater London; scale bar (3) corresponds to Washington D.C. and Manhattan

Figure 4 shows the spatial distribution of HLR in the 14 cities. In most of the cities, the human respiration is more than one order of magnitude larger than that of livestock (Additional file 1: Figures S4 and S5); thus, the spatial distribution of total HLR is dominated by the spatial distribution of humans and is very similar to that of the population (Additional file 1: Figures S1 and S4). In most of the cities, the total livestock respiration in each grid is less than 10 t C yr^− 1^. The areas around Paris have somewhat higher values, with values of almost 10 t C yr^− 1^. Moreover, the livestock respiration in eastern Beijing were greater than 60 t C yr^− 1^ and might be contributed mainly by cattle.

### Comparison with FFE

**Fig. 5:**
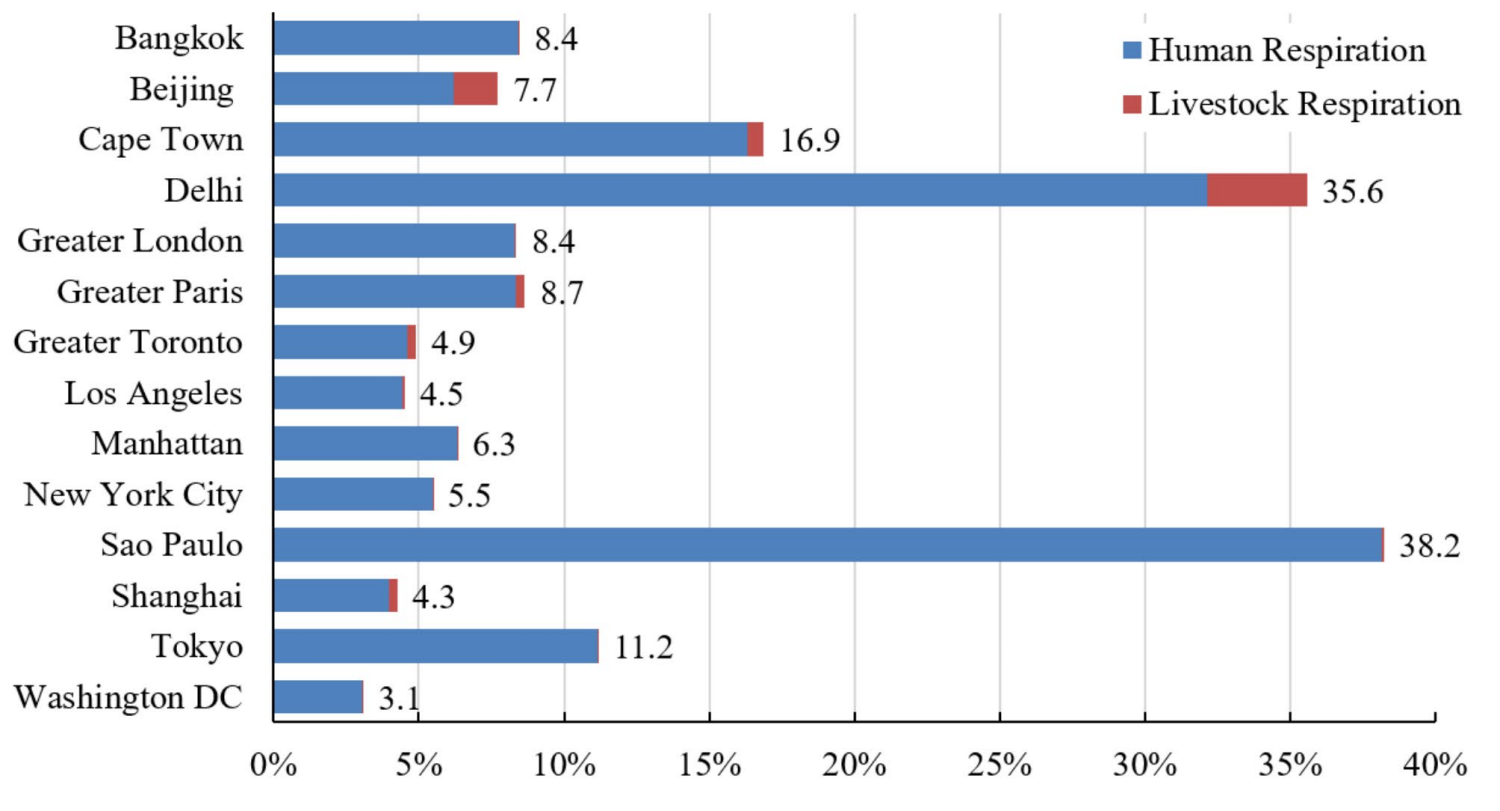
The proportions of HLR to FFE (in %). The values beside the bar show the sum value of the proportion of human and livestock respiration

Figure 5 shows the HLR compared with the FFE in cities with different areas and populations (the actual value of HLR is shown in Table 2). The HLR amounts to up to 38.2% of the FFE in Sao Paulo, almost all of which is contributed by humans. Delhi has the second highest proportion compared to FFE (nearly 35.6%), with human respiration amounting to almost 32.0% of the FFE. The high proportion to FFE in Delhi and Sao Paulo is mostly due to the relatively low amounts of FFE and their large populations. The contribution of humans and livestock in other cities is approximately 7.5% relative to higher FFE. Otherwise, the dataset of population distribution is an annual average state, which dose not reflect the diurnal variation of population in cities. In large cities, people usually live in the outskirts and commute from the suburbs to commercial areas or industrial parks. That is another reason for the lower ratio of HLR to FFE in cities such as Washington DC with a value of 3.1%.

**Fig. 6:**
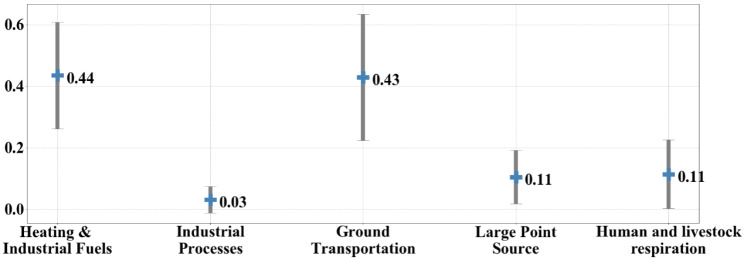
Comparison of the ratio of each sector to in-boundary FFE. The blue point represents the average ratio for the 14 cities, and the gray line is the standard error bar for the 14 cities

We also compared the ratio of total respiration to in-boundary FFE associated with other sectors (Fig. 6), including heating and industrial fuels, industrial processes, ground transportation, and large point sources, according to the results from Chen et al. [25]. Based on the average of the 14 cities, the average contribution of human and livestock respiration is comparable to that of large point sources (in-boundary) and is greater than that of industrial processes.

Moreover, we separated the source of HLR into urban and suburban. In the 14 cities overall, the HLR from urban areas is approximately 90% of the total HLR, and that from suburban areas varies from 0.4 to 32.6% (Additional file 1: Figure S6 and Table S7). In cities with relatively large proportions of HLR compared to FFE such as Delhi and Cape Town, the HLR from suburban accounts for approximately 3.3% and 3.1% of the total in-boundary FFE. As for Sao Paulo, although HLR is 36.2% of total FFE, the HLR from suburban is only 0.4% of total FFE. Furthermore, the HLR from suburban areas in Beijing accounts for up to one-third of the total HLR (2.5% of total FFE). Such a relatively high proportion is mainly contributed by the significant livestock production in suburban area, wchich accounts for 80% of the total area. Therefore, in studies with high-precision CO_2_ concentration measurements, the stations located in suburban areas of cities such as Beijing, Delhi and Cape Town should also be taken into consideration [69].

## Discussion

Carbon monitoring in urban areas has shown that the partern of human metabolism can partially explain the diurnal pattern of CO_2_ flux, as well as differences in CO_2_ flux between working days and non-working days, in densely populated urban areas [58, 59]. The research of Ciais et al. [24] shows that, in total, humans and livestock contribute 5% to FFE in urban areas globally, and the CO_2_ releases from respiration is even larger than oil burning in India and larger than FFE from gas in Chinese cities. Also, to explain the difference in the central estimate of bottom-up and top-down approaches must consider all flux contributions including the important contribution of animal respiration[23]. Thus, the HLR is not a negligible contribution in some populated cities.

### Uncertainty associated with the parameters

We compared the HLR with in-boundary FFE via a bottom-up method. The uncertainty in this study mainly comes from the uncertainty of the data source of population and livestock production, as well as the uncertainty in the assumptions of the parameters of BMR and PAL.

The BMR is affected by a variety of factors, such as age, gender, exercise, body temperature, nutritional status, or lactation [31, 60–63]. Although several equations have been developed to predict BMR [31, 40, 64] and often take into account weight, height, age, gender and other factors, considering the high-resolution data of population and livestock production used in this study, it is difficult to distinguish the above factors on this spatial resolution. Therefore, we assume that for each livestock species (mammalian livestock, poultry) individuals of different ages and genders and in different areas are assigned the same value of BMR. As for humans, we distinguished the BMR for each age-sex group in 7 global regions. In future research on more detailed CO_2_ emissions for specific urban areas, we will consider the physical differences of people in different countries and the natural environment.

In fact, BMR is only the lowest estimate of metabolic rate, and the actual respiratory intensity of each individual is related to climate and daily activity. The PAL recommended by the FAO/WHO/UNU Expert Committee in 1985 was defined in three levels: the minimum was set at 1.55 and 1.56 BMR for men and women, while the highest was defined as 2.10 and 1.82 BMR for men and women, respectively [33]. Research also showed that the PAL were not significantly different between age groups[65]. Due to lack of data, we roughly assume that the average daily activity is at the lowest level since most people do not have long-term high-intensity activity.

The carbon emissions from respiration per individual in this study are only a first-order approximation based on the above assumption. Through the weighted average of the *HR*_*h*_ for each age-sex group in 7 regions, the global averaged *HR*_*h*_ is assumed to be 89.90 kg C yr^− 1^ in this study. This result is comparable with that in other studies, which is varies over a range of 52.9–160 kg C yr^− 1^ (Additional file 1: Table S8) based on different methods or without considering different age groups and gender [7, 17, 19, 21–23, 66]. The value used by Huang et al. is much higher than other studies. As a result, their estimated annual averaged global carbon emissions from human respiration is approximately 1.2 Gt C yr^− 1^ [66] from 1990 to 2005, which is much greater than the value estimated in this study (0.54 Gt C yr^− 1^). Additionally, their estimation of livestock respiration is approximately 0.8 Gt C yr^− 1^ [66], which is comparable with this study (0.62 Gt C yr^− 1^).

### The HLR within other 118 world urban areas

In addition to the 14 selected cities, we provide a supplementary dataset on the HLR in 2010 within other 118 world urban areas larger than 100 km^2^, which were selected on the basis of being capitals or areas with large populations (greater than 1 million) or that have carbon monitoring sites, as a reference for carbon monitoring and carbon emission research (Additional file 2: Table S9). Among the 118 selected urban areas, approximately 11 urban areas have the proportions of HLR to FFE greater than 50%, and 50 urban areas have the proportions greater than 10% (Additional file 1: Figure S7 and Additional file 2: Table S9). We can see that the cities where HLR to FFE is greater than 50% are almost all the cities in countries of low income in Sub-Saharan Africa ( see Additional file 2: Table S9). In these cities, the main reason of the high ratio of HLR to FFE is the lower levels of economic development and lower fossil fuel emissions. But for the cities in high income and upper middle income countries, the ratio of HLR to FFE varies from 0.2 − 22.5% with the mean and medians of 8.2% and 6.4%, respectively. There does not appear to be a clear correlation between population density/GDP per capital and the ratio of HLR to FFE. In cities with higher population density (greater than 10,000 per km^2^), regions with higher GDP per capital tend to have lower ratio of HLR to FFE, but this is not absolutely the case. For example, Istanbul, Turkey and Bangalore, India have similar population density. In 2010, GDP per capita in Turkey (10,742 US$ in 2010) was much higher than that of India (1358 US$ in 2010), and the ratio of HLR to FFE in Istanbul (20.3%) was also higher than that of Bangalore (8.5%). In this part, due to the lack of the statistics on the GDP per capita of each city, our analysis is based on the GDP per capita of the country where the city is located. Even so, we can see that the ratio of HLR to FFE is caused by factors such as population density, urban development level, and perhaps urban cleanliness. This study provides only a global overview through first-order approximation. It is not enough to judge the ratio of HLR to FFE simply by the population density or GDP per capital of a city, and to determine whether the impact of HLR needs to be considered in the city’s carbon monitoring.

### The budget of harvested crop carbon

The processes that release CO_2_ (human and livestock respiration) and the processes of crop carbon harvest result in fluxes of CO_2_ to and from the atmosphere and constitute a part of the global CO_2_ cycle. In this section, we will briefly discuss the budget of the carbon cycle from the bottom-up approach and top-down approach.

Based on the results in the previous sections, humans and livestock together released 1.45 Gt C in 2010 (0.64 and 0.81 Gt C, respectively). Forage grass is a local feed resource of horses, sheep and goats, as well as a portion of the feed for poultry, pigs and housed cattle in developing countries [18]. We follow the assumption of Ciais et al. that only 20% of poultry, pigs and cattle in developing countries received grain-based feeds, whereas 100% of these livestock in developed countries received grain-based feeds. Other livestock received local feed resources in our assumption. Based on the livestock production in each country from the FAO [55], we find that the carbon emissions from livestock utilizing local feed sources was approximately 0.64 Gt C (approximately 79% of total livestock respiration) in 2010. Hence, the carbon released by livestock respiration comes from grain-based feeds is approximately 0.17 Gt C in 2010 (about 21% of total livestock respiration).

Here, we also considered food waste. According to the FAO, approximately one-third of food produced by humans is lost or wasted globally every year [67]. Crop production is estimated to have been approximately 1.50 Gt C in 2010 based on crop production from the FAO [68], which is slightly larger than the human and livestock consumption estimated in this study.

The budget of harvested crop carbon is constructed as follows:

4 $$P-W={R}_{human}+{R}_{livestock}\times 0.21$$

where *P* is crop carbon production via the top-down modeling method, *W* is the lost and wasted crop production, *R*_*human*_ and *R*_*livestock*_ are the carbon releases from human and livestock respiration estimated by the bottom-up approach, and only 0.21 of *R*_*livestock*_ comes from grain-based feeds. Based on the above discussion, we estimate that *W* is approximately 0.5 Gt C. Excluding food loss and waste, crop production used for metabolization is approximately 1.00 Gt C, which has the same magnitude and is comparable to human and livestock consumption (0.81 Gt C, excluding livestock feeding on local forage grass).

In addition, we should note that the carbon released by livestock respiration in 14 cities also includes local feed sources. In particular, in Delhi and Beijing, the percentages of livestock respiratory carbon are 3.4% and 1.5% relative to fossil fuel. Considering livestock fed with local feed, livestock respiration should have less influence on city carbon emissions than our estimation with in cities.

## Conclusion

In this study, we used the global population and livestock production, as well as the parameter of BMR, to estimate the amount of carbon released by human and livestock respiration from 1990 to 2014. Then, we calculated the carbon emissions from human and livestock respiration in 14 of the world’s largest cities and compared them with the in-boundary FFE. The results showed that the proportion of total carbon released from humans and livestock is approximately 5–10% relative to in- boundary FFE in most of the 14 typical cities. In cities such as Delhi, Sao Paulo and Cape Town, humans and livestock contribute up to 38.2% relative to FFE. In studies monitoring FFE from ground stations or satellites, neglicting human/livestock emissions could overestimate the in-boundary FFE [21] or leave ambiguity in the evaluation of the FFE trend [24]. In addition, approximately 90% of the respiratory carbon is released in the urban areas of most cities, while the suburban HLR has a noteable contribution compared to FFE in Beijing, Delhi and Cape Town. This means that the setup of carbon monitoring sites should not neglect suburban areas in those cities. Further more, the results in suburban areas also helps to analyse the vertical distribution of CO_2_ in the boundary layer, and provide data for validating transport models[69]. We should also note that this study used global unified values for BMR for livestock and PAL for all species. To estimate the carbon from human and livestock respiration more accurately and to compare it with FFE, it is better to adopt parameters appropriate for regional or national climate and livestock feeding conditions.

## Electronic supplementary material

Below is the link to the electronic supplementary material.

Supplementary Material 1: Table S9 The HLR within 118 worldurban areas. Notes: The 118 world urban areas are selected onthe basis of area larger than 100 km2, being capitals, with populationsgreater than 1 million or have carbon monitoring sites.

Supplementary Material 2: Supplementary tables and figures.

## Data Availability

The datasets supporting the conclusions of this article are included within the article and its additional files. High-resolution data are available upon request to corresponding author.
