# Supplementary material for: The impact of human and livestock respiration on CO2 emissions from 14 global cities
Source: Carbon Balance Manag. 2022 Nov 3;17:17. doi: 10.1186/s13021-022-00217-7 (PMC9635100; doi:10.1186/s13021-022-00217-7)
Supplement: Supplementary file 2 — Supplementary Material 2: Supplementary tables and figures. [file 13021_2022_217_MOESM2_ESM.docx]

# Supplementary Information

## Method:

The BMR for individuals of both sexes was given at different body weights throughout the age range of infancy and adults. Once we have the mean weight for each region, we can obtain the mean BMI corresponding to the mean weight by looking up to the table of predicted daily BMR from FAO (Table S1) [1]. However, the mean weight statistics by age and gender for global countries or regions is rarely seen in existing global databases.

Thus, we use the parameter of BMI, which is a value derived from the body mass (weight) and the square of body height of a person. The BMI and body height are obtained from the research in recent years. In this study, the mean BMI and mean height in age 5-19 in global 7 regions are obtained from the website of NCD Risk Factor Collaboration (NCD-RisC, https://www.ncdrisc.org/data-downloads.html) [2,3]. The BMI and body height of adults aged more than 20 years are from WHO SURF2 report [4] and NCD-RisC [5]. The body weight we used in this study is show in Table S3.

Table S1: The BMR of 6 age-sex groups in 7 global regions (unit: MJ).

| Region | ages 0-9, female | ages 0-9, Male | ages 10-19, female | ages 10-19, Male | ages 20+, female | ages 20+, Male |
| --- | --- | --- | --- | --- | --- | --- |
| South Asia | 3.7 | 3.7 | 5.2 | 5.5 | 6.6 | 6.3 |
| East Asia & Pacific | 4.2 | 4.2 | 5.7 | 6.2 | 7.0 | 6.8 |
| Sub-Saharan Africa | 4.0 | 3.9 | 5.6 | 5.8 | 7.0 | 6.3 |
| Latin America & Caribbean | 4.4 | 4.4 | 5.8 | 6.3 | 7.1 | 6.7 |
| Europe & Central Asia | 4.4 | 4.4 | 5.9 | 6.5 | 7.3 | 6.8 |
| Middle East & North Africa | 4.2 | 4.2 | 5.8 | 6.2 | 6.8 | 6.3 |
| North America | 4.6 | 4.6 | 6.0 | 6.8 | 7.0 | 7.0 |

Table S2: The O_2_ consumption of per person for 6 age-sex groups in 7 global regions (unit: L O_2_ day^-1^).

| Region | ages 0-9, female | ages 0-9, Male | ages 10-19, female | ages 10-19, Male | ages 20+, female | ages 20+, Male |
| --- | --- | --- | --- | --- | --- | --- |
| South Asia | 3.7 | 3.7 | 5.2 | 5.5 | 6.6 | 6.3 |
| East Asia & Pacific | 4.2 | 4.2 | 5.7 | 6.2 | 7.0 | 6.8 |
| Sub-Saharan Africa | 4.0 | 3.9 | 5.6 | 5.8 | 7.0 | 6.3 |
| Latin America & Caribbean | 4.4 | 4.4 | 5.8 | 6.3 | 7.1 | 6.7 |
| Europe & Central Asia | 4.4 | 4.4 | 5.9 | 6.5 | 7.3 | 6.8 |
| Middle East & North Africa | 4.2 | 4.2 | 5.8 | 6.2 | 6.8 | 6.3 |
| North America | 4.6 | 4.6 | 6.0 | 6.8 | 7.0 | 7.0 |

Table S3: The body weight of per person for 6 age-sex groups in 7 global regions (unit: Kg).

| Region | ages 0-9, female | ages 0-9, Male | ages 10-19, female | ages 10-19, Male | ages 20+, female | ages 20+, Male |
| --- | --- | --- | --- | --- | --- | --- |
| South Asia | 18.1 | 18.2 | 37.9 | 39.4 | 61.0 | 68.2 |
| East Asia & Pacific | 23.4 | 23.2 | 47.4 | 50.0 | 67.4 | 80.8 |
| Sub-Saharan Africa | 20.7 | 19.4 | 44.1 | 42.8 | 66.7 | 68.1 |
| Latin America & Caribbean | 24.3 | 24.8 | 48.9 | 52.1 | 69.2 | 78.7 |
| Europe & Central Asia | 24.9 | 25.2 | 50.8 | 54.6 | 73.1 | 81.2 |
| Middle East & North Africa | 23.1 | 23.1 | 50.3 | 50.3 | 62.6 | 66.9 |
| North America | 26.9 | 27.2 | 54.8 | 59.9 | 76.6 | 90.2 |

Reference:

1. J.V.G.A.Durnin. Basal metabolic rate in man [Internet]. Rome: FAO; 1981 Oct. Available from: http://www.fao.org/3/M2845E/M2845E00.htm

2. Abarca-Gómez L, Abdeen ZA, Hamid ZA, Abu-Rmeileh NM, Acosta-Cazares B, Acuin C, et al. Worldwide trends in body-mass index, underweight, overweight, and obesity from 1975 to 2016: a pooled analysis of 2416 population-based measurement studies in 128·9 million children, adolescents, and adults. The Lancet. 2017;390:2627–42.

3. Rodriguez-Martinez A, Zhou B, Sophiea MK, Bentham J, Paciorek CJ, Iurilli ML, et al. Height and body-mass index trajectories of school-aged children and adolescents from 1985 to 2019 in 200 countries and territories: a pooled analysis of 2181 population-based studies with 65 million participants. The Lancet. 2020;396:1511–24.

4. WHO Global Infobase Team. The SuRF report 2. Surveillance of chronic disease risk factors: country-level data and comparable estimates. Geneva: World Health Organization; 2005.

5. NCD Risk Factor Collaboration (NCD-RisC). A century of trends in adult human height. eLife. 2016;5:e13410.

Table S4: Original parameters of BMR for all kinds of species

| Taxon | Body weight  (g) | BMR  (ml O_2_ g^-1^ h^-1^) | References |
| --- | --- | --- | --- |
| horse | 260000 | 0.25 | M. A. Elgar and P. H. Harvey (1987) |
| pig | 75000 | 0.11 |  |
| cattle/buffalo | 272000 | 0.17 |  |
| goat | 36000 | 0.19 |  |
| sheep | 30000 | 0.34 |  |
| poultry | 862.3^*^ | 2.50^*^ | B. M. Freeman (1963) |

* Body weight is the average of different breeding ages and gender. The BMR is the average of different ages.

Table S5: Comparison of cattle production from GLW and national census.

| cattle | GLW | census | source |
| --- | --- | --- | --- |
| Beijing (10000 head) | 547.0 | 31.8 | National Agriculture Census in China |
| Shanghai (10000 head) | 6.4 | 6.8 | National Agriculture. Census in China |
| Delhi (10000 head) | 9.9 | 8.2 | 19 Livestock Census-2012 All India Report |
| São Paulo (10000 head) | 413.0 | 500.0 | Produção da Pecuária Municipal - 2014 source, Istituto Brasileiro de Geografia e Estatistica, |

* To be consistent with GLW, the census statistic data comes from national census 2010.

Table S6: Comparison of livestock production in Beijing

| Production (10000 head) | GLW | census |
| --- | --- | --- |
| pig | 219.0 | 494.0 |
| chicken/duck | 1074.0 | 11774.0 |
| sheep/goat | 158.4 | 60.6 |
| cattle | 547.0 | 31.8 |
| horse | 0.3 | 0.2 |

* The census statistic data coms from National Bureau of Statistics of China 2010.

Table S7 Compare the carbon release from respiration within urban and suburban areas within the 14 cities of focus.

| City or metropolitan region | City area | | Total respiration | | Human | | Livestock | |
| --- | --- | --- | --- | --- | --- | --- | --- | --- |
|  | Urban (%) | Suburban (%) | Urban (%) | Suburban (%) | Urban (%) | Suburban (%) | Urban (%) | Suburban (%) |
| Bangkok | 75.0 | 25.0 | 93.3 | 6.7 | 93.5 | 6.5 | 56.9 | 43.1 |
| Beijing | 20.1 | 79.9 | 67.4 | 32.6 | 81.3 | 18.7 | 15.0 | 85.0 |
| Cape Town | 30.3 | 69.7 | 81.5 | 18.5 | 83.0 | 17.0 | 30.7 | 69.3 |
| Delhi | 84.2 | 15.8 | 90.8 | 9.2 | 91.3 | 8.7 | 86.0 | 14.0 |
| Greater London | 93.8 | 6.2 | 99.3 | 0.7 | 99.4 | 0.6 | 89.6 | 10.4 |
| Greater Paris | 27.4 | 72.6 | 88.3 | 11.7 | 89.7 | 10.3 | 49.4 | 50.6 |
| Greater Toronto | 24.8 | 75.2 | 79.1 | 20.9 | 82.4 | 17.6 | 12.1 | 87.9 |
| Los Angeles | 35.7 | 64.3 | 96.0 | 4.0 | 96.7 | 3.3 | 33.4 | 66.6 |
| Manhattan | 72.5 | 27.5 | 91.4 | 8.6 | 91.4 | 8.6 | 69.6 | 30.4 |
| New York | 91.5 | 8.5 | 98.3 | 1.7 | 98.3 | 1.7 | 87.5 | 12.5 |
| Sao Paulo | 69.8 | 30.2 | 99.0 | 1.0 | 99.1 | 0.9 | 71.5 | 28.5 |
| Shanghai | 51.3 | 48.7 | 85.4 | 14.6 | 88.0 | 12.0 | 45.5 | 54.5 |
| Tokyo | 71.8 | 28.2 | 97.5 | 2.5 | 97.5 | 2.5 | 77.5 | 22.5 |
| Washington DC | 92.1 | 7.9 | 99.6 | 0.4 | 99.6 | 0.4 | 94.0 | 6.0 |

Table S8. CO_2_ emission from individual from literatures.

| Value  kg C yr^-1^ | Notes of source parameter | reference |
| --- | --- | --- |
| 52.96 ± 0.43 | For all ages and genders. | West, 2009 |
| 99.5 | Typically 1 kg CO_2_ day^-1^ | Bréon et al., 2014 |
| 79.0 |  | Zhao et al., 2014 |
| 92.7 | Average of 254 g C day^-1^. | Gurney et al., 2017 |
| 76.3 | 8.87 mg CO_2_ s^-1^ per person. | Christen et al., 2010 |
| 138.0 | 31.5 mol CO_2_ day^-1^. Assuming an average person weighs 70 kg, metabolic rate is about 2 times of resting, and only metabolize carbohydrate. | Koerner and Klopatek, 2002 |
| 160.1 | 1.61 kg of CO_2_ day^-1^ | Huang et al., 2018 |


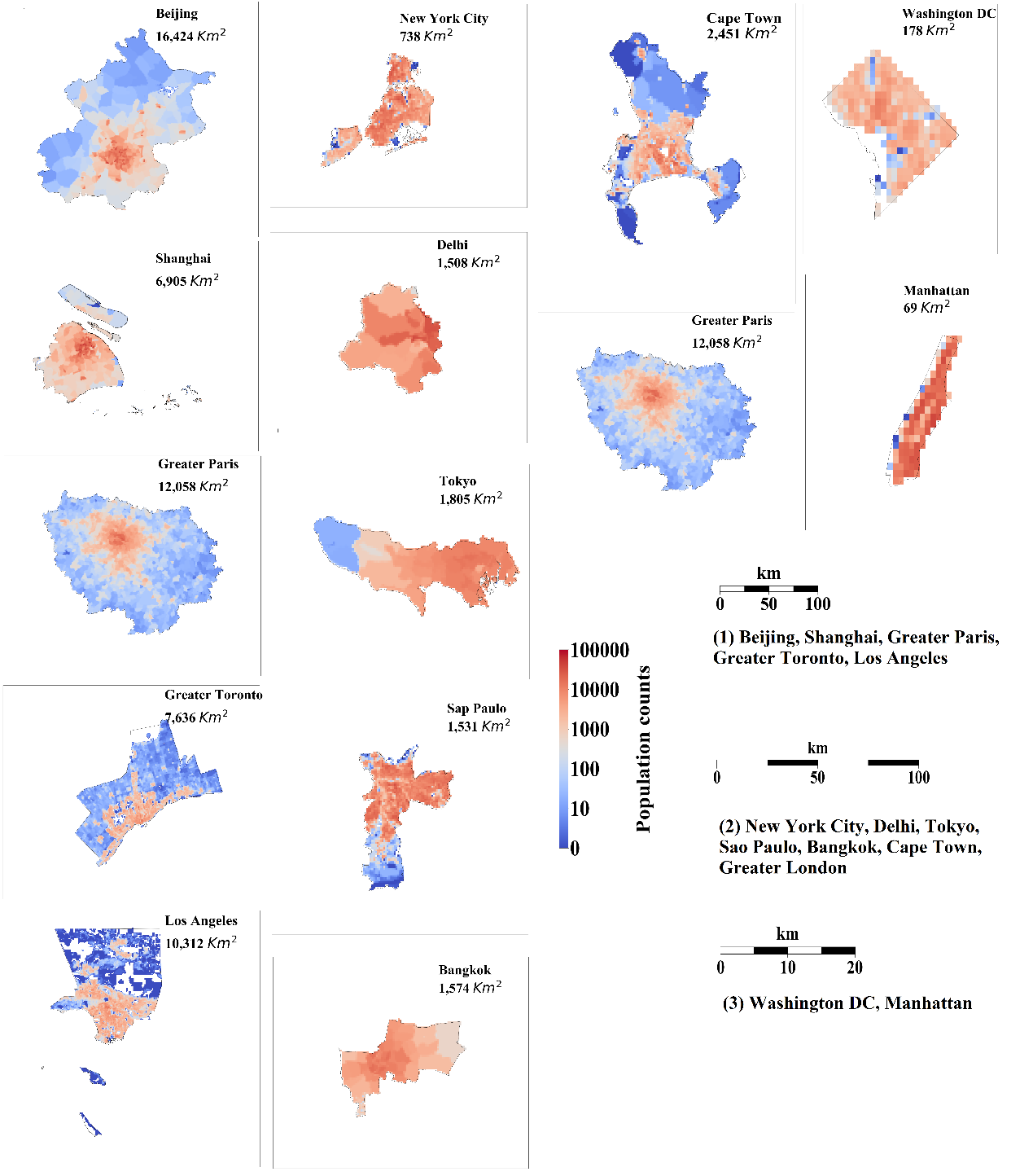


Figure S1. Population counts in 14 cities and metropolitan regions in 2010 at a resolution of 1km based on the GPW high-resolution datasets. Three scale bars were included in this distribution. Scale bar (1) corresponds to Beijing, Shanghai, Greater Paris, Greater Toronto and Los Angeles; scale bar (2) to New York City, Delhi, Tokyo, Sao Paulo, Bangkok, Cape Town and Greater London; scale bar (3) to Washington D.C. and Manhattan.


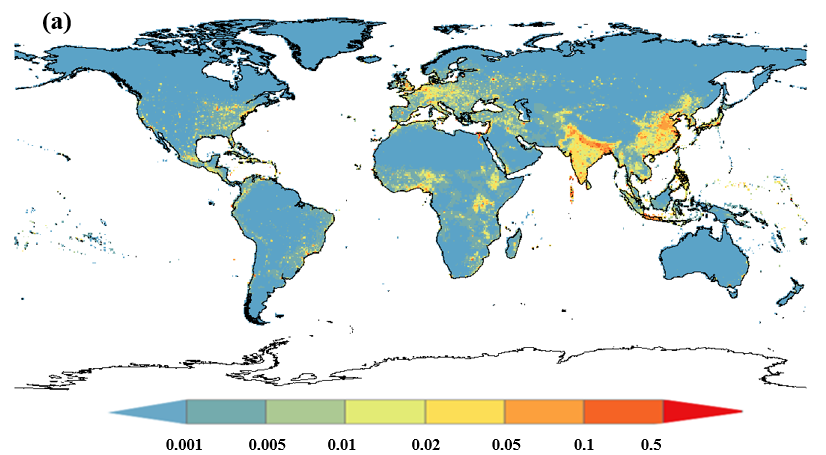

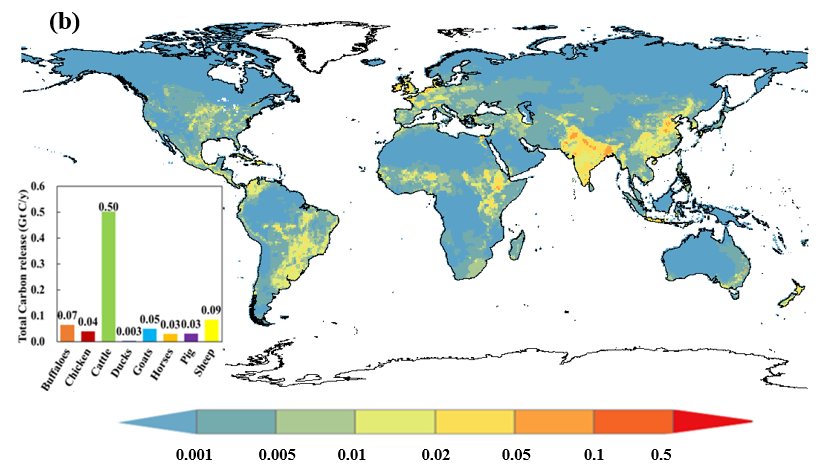


Figure S2. The spatial distribution of carbon release from (a) human and (b) livestock respiration (kg C m^-2^ yr^-1^). The bar graph in (b) shows the global total carbon output (Gt C yr^-1^) from each species of livestock in 2010.


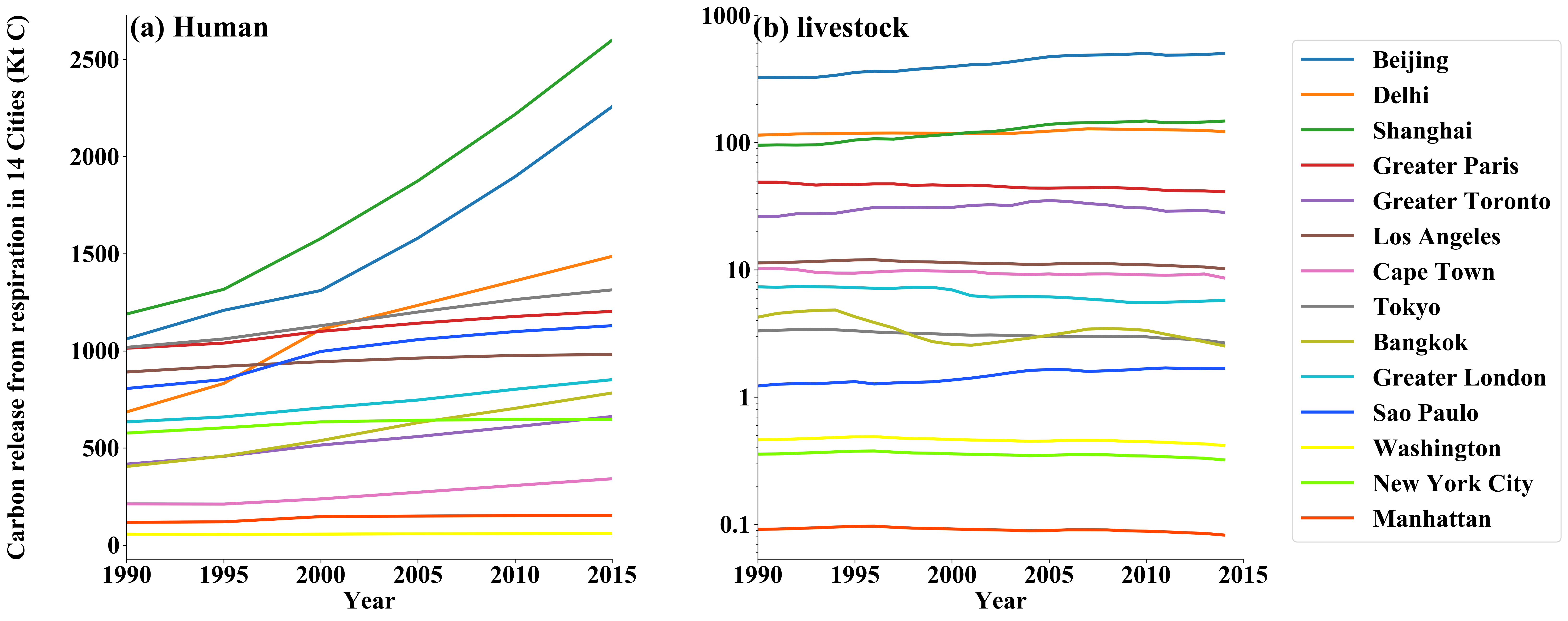


Figure S3. Variation of carbon emission from (a) human and (b) livestock respiration in 14 cities (g C m^2^ yr^-1^). The value of carbon emission from animal is presented in with a logarithmic scale along the y-axis.


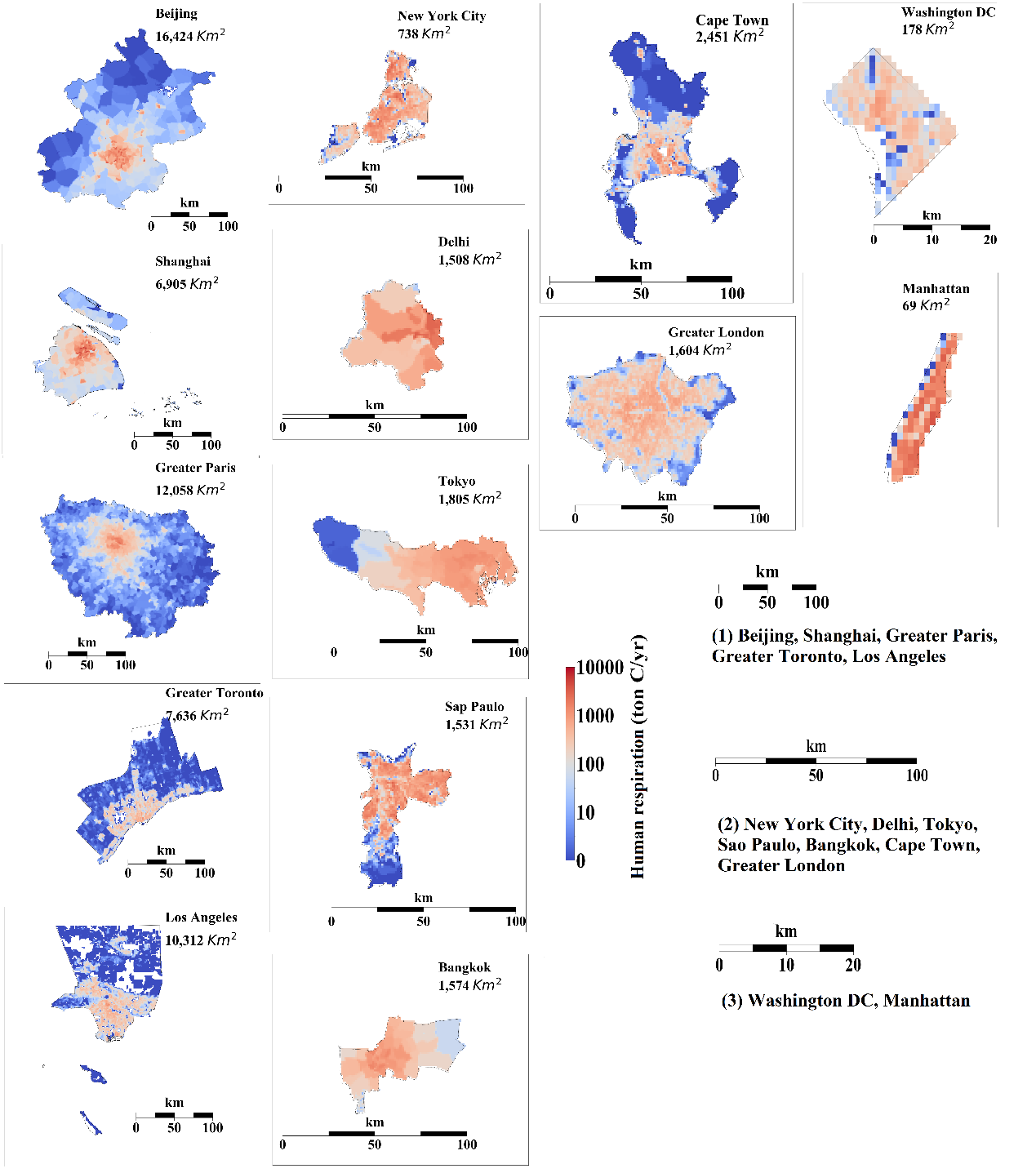


Figure S4. Carbon released from human respiration in 14 cities and metropolitan regions in 2010. Three scale bars were included in this distribution. Scale bar (1) corresponds to Beijing, Shanghai, Greater Paris, Greater Toronto and Los Angeles; scale bar (2) to New York City, Delhi, Tokyo, Sao Paulo, Bangkok, Cape Town and Greater London; scale bar (3) to Washington D.C. and Manhattan.


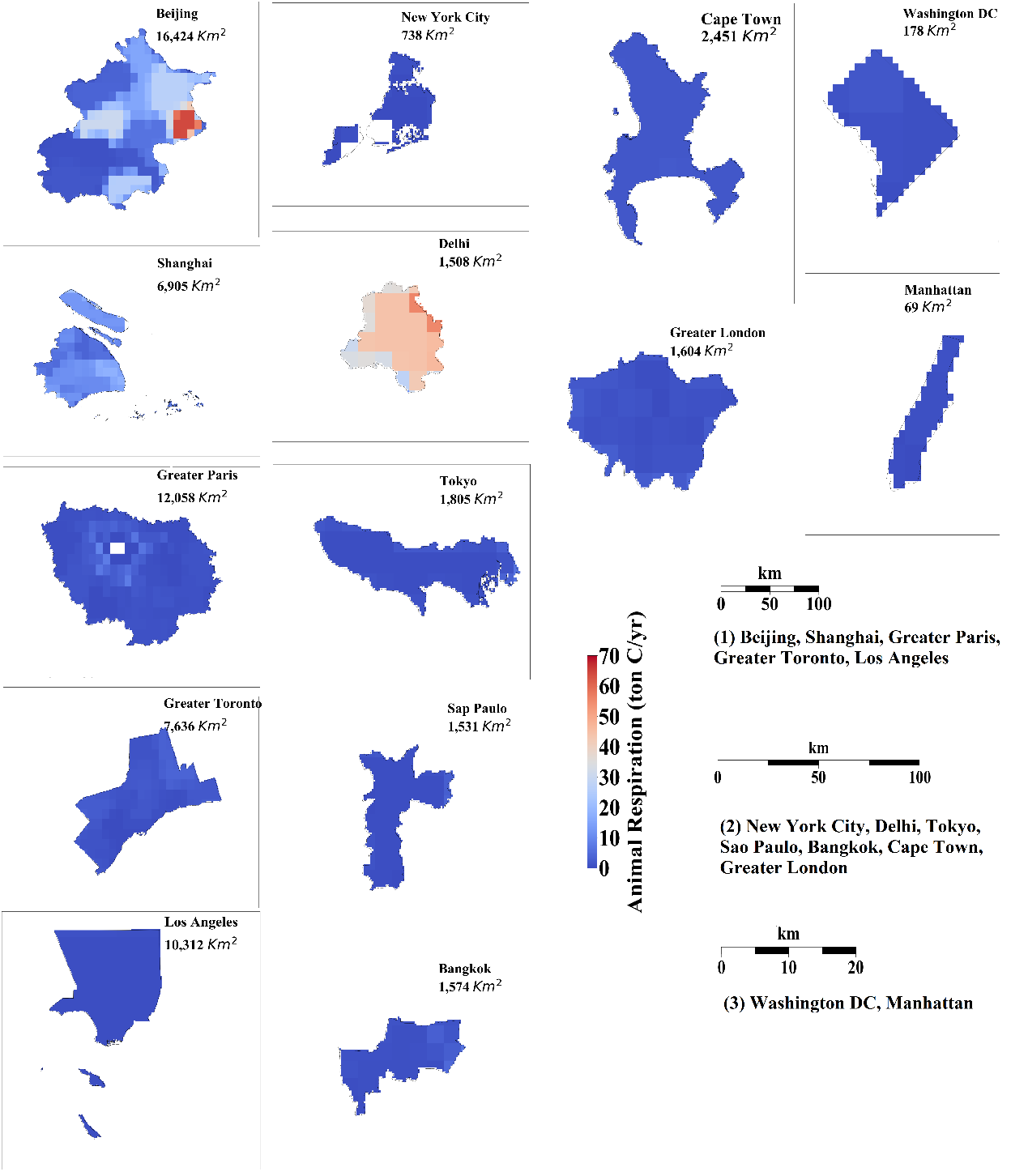


Figure S5. Carbon released from animal respiration in 14 cities and metropolitan regions in 2010. Three scale bars were included in this distribution. Scale bar (1) corresponds to Beijing, Shanghai, Greater Paris, Greater Toronto and Los Angeles; scale bar (2) to New York City, Delhi, Tokyo, Sao Paulo, Bangkok, Cape Town and Greater London; scale bar (3) to Washington D.C. and Manhattan.


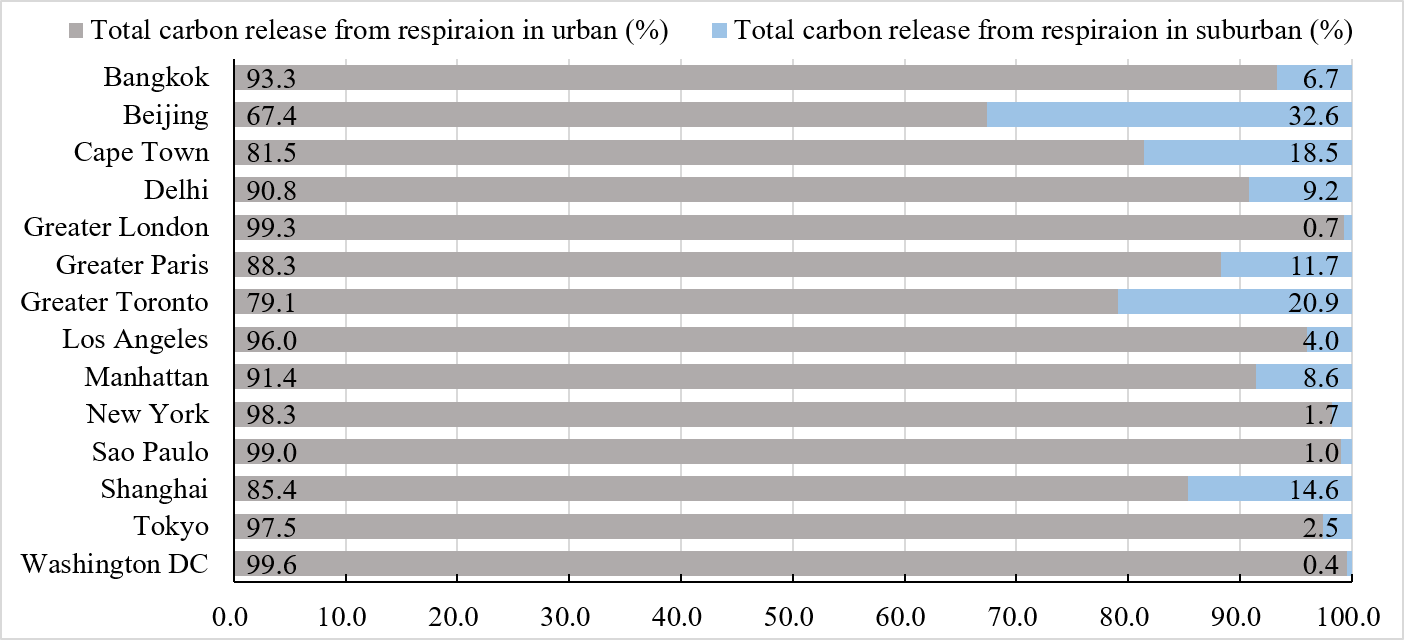


Figure S6. The proportions of total HLR in urban and suburban areas (in %). The values beside the left side of the bar are the proportions of CO_2_ from urban areas, and the values beside the right side of the bar are the proportions of CO_2_ from suburban areas.


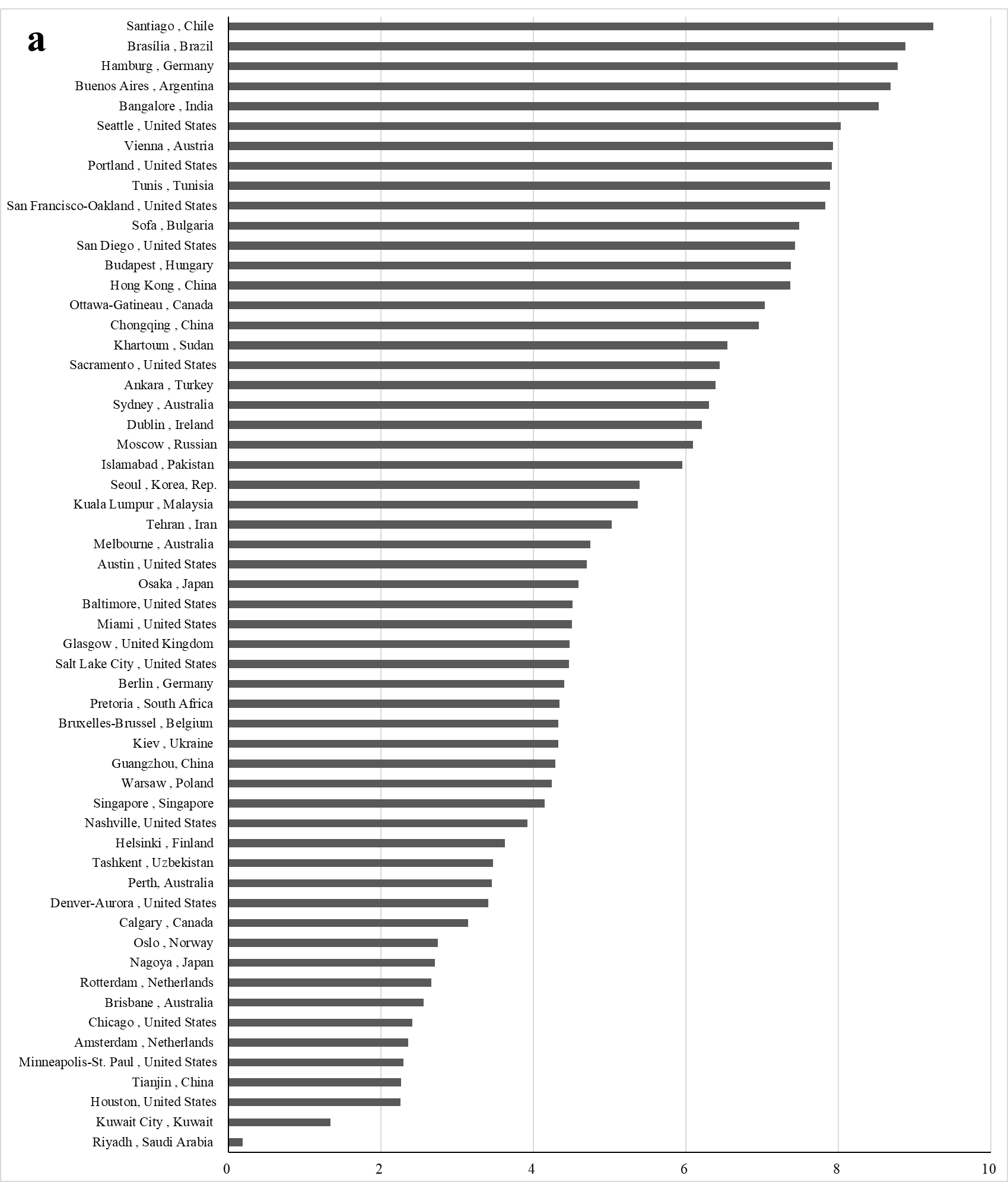

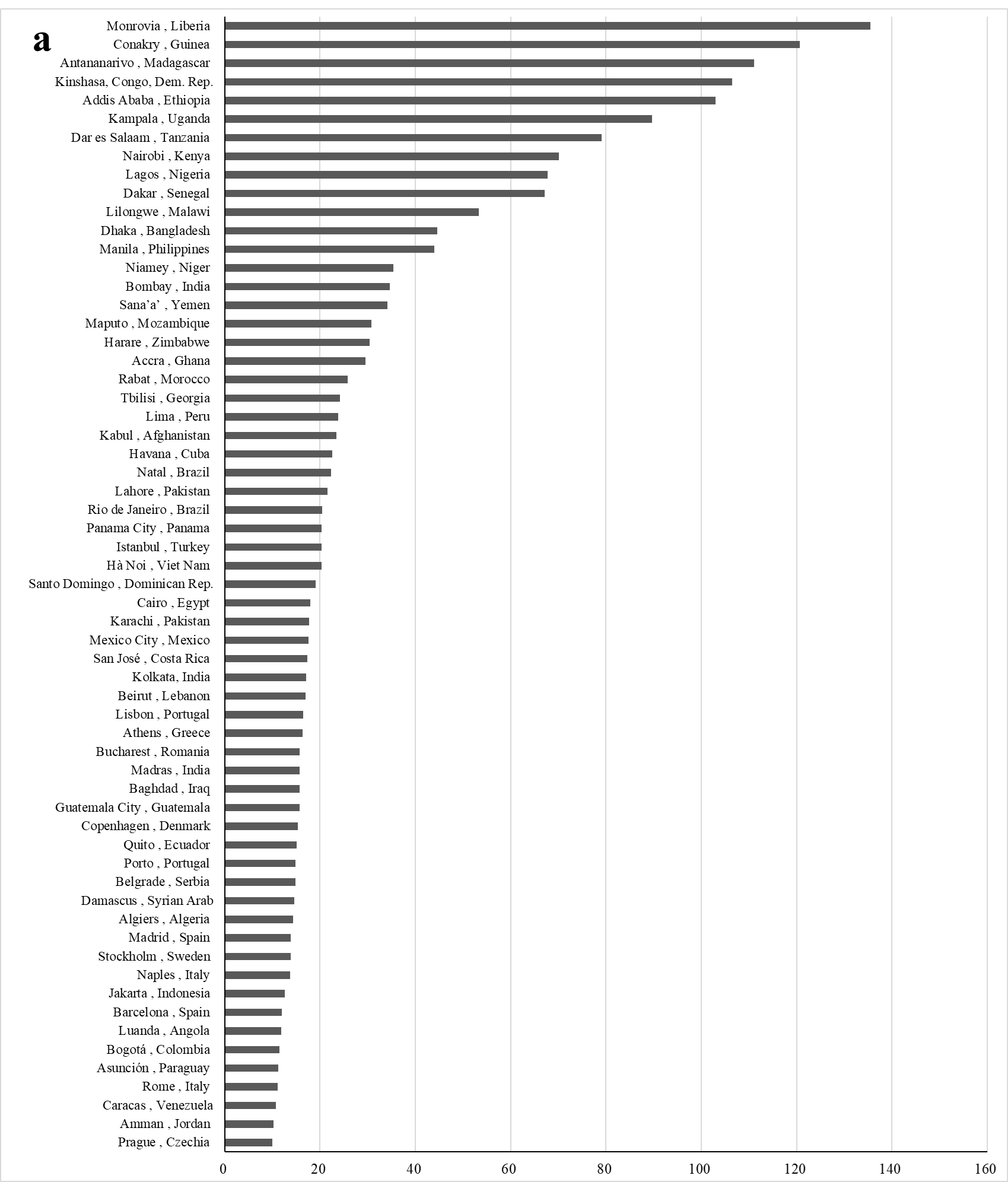


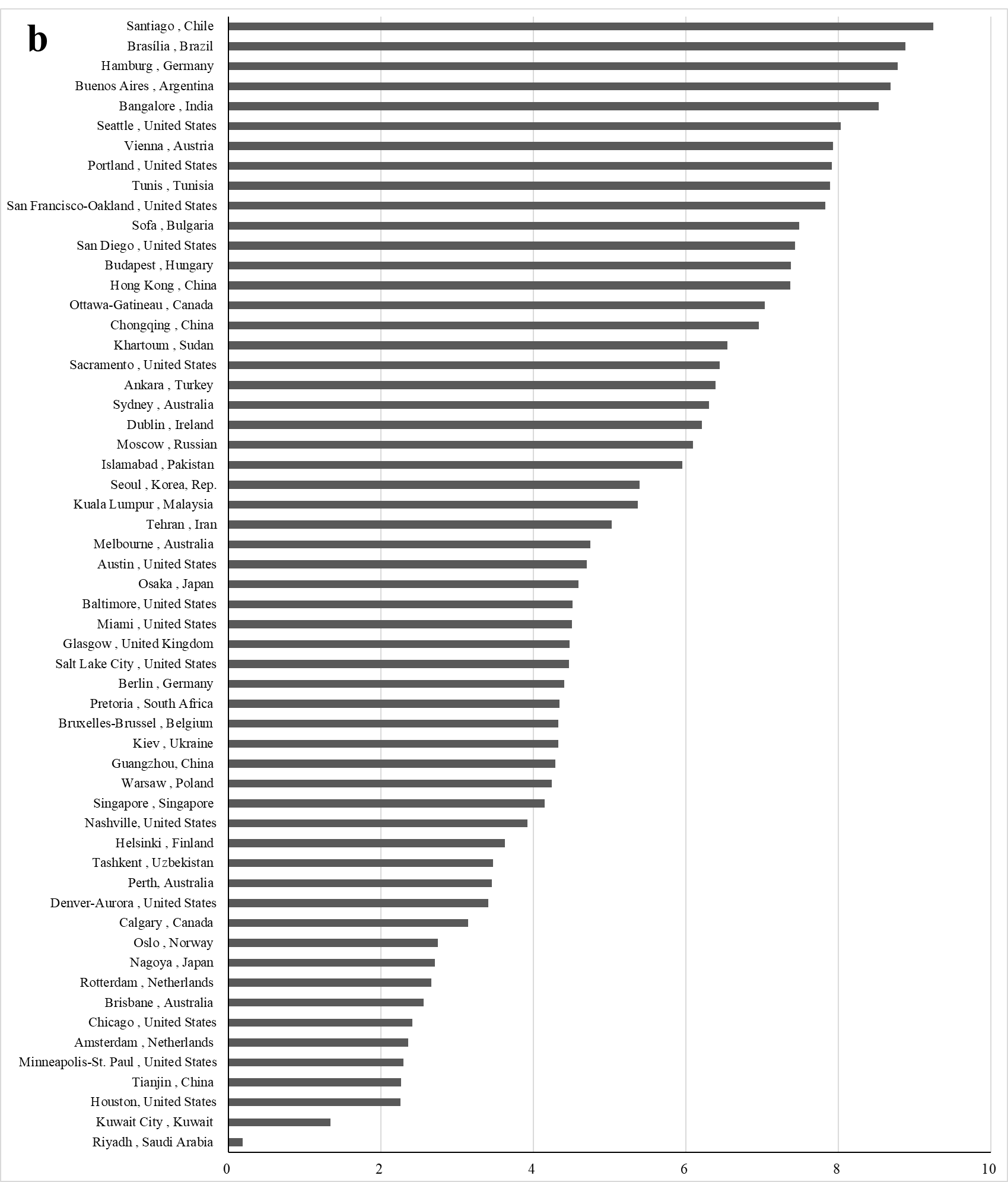
 Figure S7. The ratio of total HLR to FFE in the 118 world urban areas (in %). (a) The urban areas with ratio larger than 10%. (b) The urban areas with ratio less than 10%.
